# Proscillaridin A Promotes Oxidative Stress and ER Stress, Inhibits STAT3 Activation, and Induces Apoptosis in A549 Lung Adenocarcinoma Cells

**DOI:** 10.1155/2018/3853409

**Published:** 2018-01-11

**Authors:** Amara Maryam, Tahir Mehmood, Qiulong Yan, Yongming Li, Muhammad Khan, Tonghui Ma

**Affiliations:** ^1^College of Basic Medical Sciences, Dalian Medical University, Dalian, Liaoning 116044, China; ^2^Department of Biochemistry and Molecular Biology, Dalian Medical University, Dalian, Liaoning 116044, China

## Abstract

Cardiac glycosides are natural compounds used for the treatment of cardiovascular disorders. Although originally prescribed for cardiovascular diseases, more recently, they have been rediscovered for their potential use in the treatment of cancer. Proscillaridin A (PSD-A), a cardiac glycoside component of *Urginea maritima*, has been reported to exhibit anticancer activity. However, the cellular targets and anticancer mechanism of PSD-A in various cancers including lung cancer remain largely unexplored. In the present study, we found that PSD-A inhibits growth and induces apoptosis in A549 lung adenocarcinoma cells. The anticancer activity of PSD-A was found to be associated with the activation of JNK, induction of ER stress, mitochondrial dysfunction, and inhibition of STAT3 activation. PSD-A induces oxidative stress as evidenced from ROS generation, GSH depletion, and decreased activity of TrxR1. PSD-A-mediated ER stress was verified by increased phosphorylation of eIF2*α* and expression of its downstream effector proteins ATF4, CHOP, and caspases-4. PSD-A triggered apoptosis by inducing JNK (1/2) activation, increasing bax/bcl-2 ratio, dissipating mitochondrial membrane potential, and inducing cleavage of caspases and PARP. Further study revealed that PSD-A inhibits both constitutive and inducible STAT3 activations and decreases STAT3 DNA-binding activity. Moreover, PSD-A-mediated inhibition of STAT3 activation was found to be associated with increased SHP-1 expression, decreased phosphorylation of Src, and binding of PSD-A with STAT3 SH2 domain. Finally, STAT3 knockdown by shRNA inhibited growth and enhanced apoptotic efficacy of PSD-A. Taken together, the data suggest that PSD-A could be developed into a potential therapeutic agent against lung adenocarcinoma.

## 1. Introduction

Lung cancer is the most commonly diagnosed malignancy and the 1st leading cause of cancer-related fatalities worldwide [1]. Lung cancer is broadly divided into main categories: small cell lung cancer (SCLC) and nonsmall cell lung cancer (NSCLC) [2] NSCLC which is composed of three predominant subtypes: adenocarcinoma, squamous cell carcinoma, and large cell carcinoma, is the most common cancer which accounts for approximately 80–85% of all lung cancer cases [3]. Despite improvements in surgical techniques and availability of new highly targeted therapies such as EGFR-directed tyrosine kinase inhibitors (TKIs), the prognosis of NSCLC remains still very poor with a 5-year survival rate about 15% which is only 5% higher than the survival rate 40 years ago [4]. Exploring novel therapeutic agents and their anticancer mechanisms is, therefore, necessary for improving the outcome of lung cancer treatment.

At present, platinum-based chemotherapeutics such as cisplatin and carboplatin are the first-line treatment for NSCLC patients followed by second-line chemotherapy with docetaxel and/or EGFR-directed TKIs such as gefitinib and erlotinib [5, 6]. However, drug resistance has become the major limitation of these drugs [7, 8]. Signal transducer and activator of transcription 3 (STAT3) is an important transcription factor that plays a key role in multiple cellular functions such as cell growth, survival, differentiation, metabolism, host defense, and immunoregulation. While in normal cells, STAT3 activation is strictly controlled; in cancer cells, it is persistently activated. Accumulating evidence from various studies strongly implicates the role of aberrantly active STAT3 in tumorigenesis, drug resistance, and metastasis of various human cancers including NSCLC [9–11]. Inhibition of STAT3 activation by genetic and pharmacological approaches has been shown to suppress tumor growth and enhance the sensitivity of clinical drugs in various *in vitro* and *in vivo* models [12–14]. Recent research has shown that currently available chemotherapeutic drugs for NSCLC induce STAT3 activation [9, 15, 16], suggesting that STAT3 may play an important role in tumor resistance to prevailing chemotherapy in NSCLC. Exploring novel cytotoxic agents with STAT3 suppressive activity might hold a greater potential to reduce mortality and improve the outcome of NSCLC treatment.

**Figure 1:**
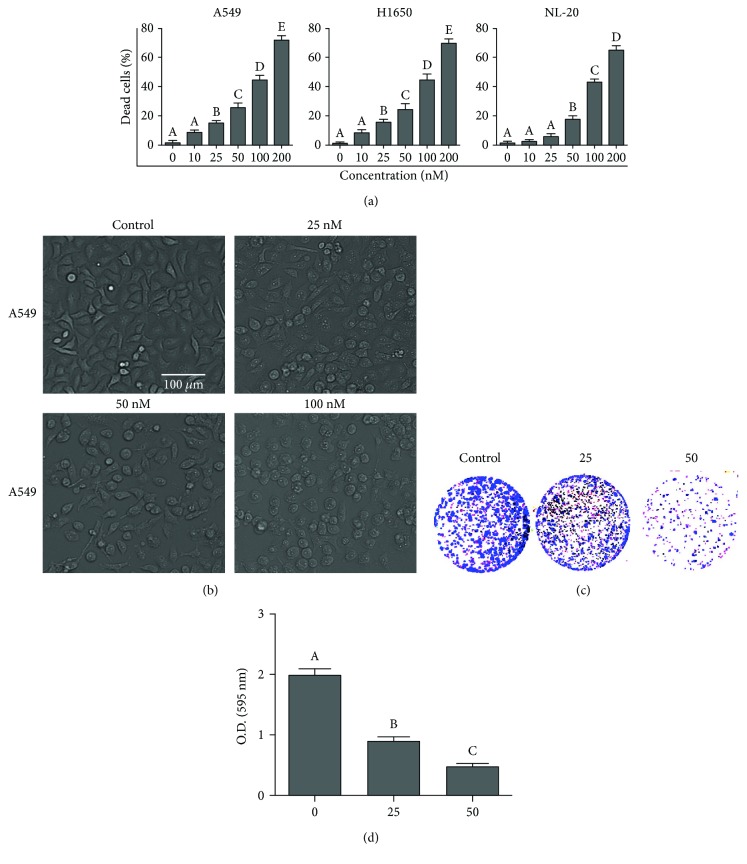
PSD-A induces cytotoxicity in A549 lung cancer cells. (a) A549, H1650, and NL-20 cells were treated with indicated concentrations of PSD-A for 24 h, and live and dead cells were quantified by TBE assay. (b) A549 cells were treated with indicated concentrations of PSD-A for 24 h, and cell morphological changes were observed under a phase contrast microscope. Scale bar 100 *μ*m. (c) Cells treated with indicated concentration of PSD-A for 24 h were seeded into 6-well plates and allowed to grow into colonies for 7 days. The colonies were fixed, stained with crystal violet, and photographed. (d) To quantify the effect of PSD-A on cell proliferation, stain was dissolved in methanol and absorption was checked at 595 nm. Data from (a, c) are expressed as mean ± SEM (*n* = 3). Columns not sharing the same superscript letters differ significantly (*P* < 0.05).

Cardiac glycosides are natural compounds which contain a steroid nucleus with an unsaturated lactone ring at position 17 (C-17) and a sugar moiety at position 3 (C-3). Based on the lactone ring, they can be categorized into two main groups: those containing a 5-membered lactone ring are called cardenolides while those containing a 6-membered lactone ring are called bufadienolides. Cardiac glycosides have long been used to treat heart failure. The cardiotonic effect of cardiac glycosides has been identified to be mediated by their ability to selectively inhibit Na^+^/K^+^-ATPase pump [17, 18]. Although originally prescribed to treat cardiac congestions and cardiac arrhythmias, more recently, cardiac glycosides have been rediscovered for their potential use in the treatment of cancer. Since the first epidemiologic evidence reported for anticancer activity of cardiac glycoside *Digitalis* in 1980, several studies have been conducted to explore the anticancer activity of cardiac glycosides. The published data indicate that cardiac glycosides exhibit significant anticancer activity against a wide range of human cancer types both *in vitro* and *in vivo* through multiple mechanisms including inhibition of proliferation, induction of apoptosis, and augmentation of chemotherapy. More importantly, it has been found that the doses of cardiac glycosides that are active against cancer cells are even lower than those found in the plasma of heart patients treated with cardiac glycosides, suggesting that cardiac glycosides exert anticancer activity at nontoxic concentrations [17, 19]. These clinical observations highlight the importance and support the potential use of these drugs for cancer treatment. In the present study, we have shown that PSD-A (Figure 1(a)), a bufadienolide cardiac glycoside component of *Urginea maritima* [20], inhibits growth and induces apoptosis in A549 lung adenocarcinoma cells. Moreover, inhibition of STAT3 activation and induction of oxidative stress and ER stress by PSD-A in the present study disclose the previously unrecognized mechanisms.

## 2. Materials and Methods

### 2.1. Antibodies and Reagents

A549 lung adenocarcinoma cells were obtained from American Type Culture Collection (Manassas VA) while 293FT cells were obtained from Shanghai Chinese Academy of Science. Packaging vectors and shRNA oligonucleotides were obtained from Addgene (USA) and Thermo Fisher Scientific (USA), respectively. PSD-A (purity > 98%) was purchased from EXTRASYNTHESE (Genay, France). Dulbecco's Modified Eagle's Medium (DMEM) and fatal bovine serum (FBS) were obtained from Gibco (Eggenstein, Germany). Penicillin and streptomycin were purchased from Solarbio Co. Ltd. (Beijing, China). Annexin V-FITC apoptosis detection kit, ROS assay kit, mitochondrial membrane potential assay kit with JC-1, Crystal violet staining solution, caspase-4 activity assay kit, N-acetyl-L-cysteine (NAC), Fluo-3 AM, and trypan blue were obtained from Beyotime Biotechnology (Haimen, Jiangsu, China). 3-(4,5-Dimethylthiazol-2-yl)-2,5-diphenyltetrazolium bromide (MTT), propidium iodide (PI), calcein AM, dimethyl sulfoxide (DMSO), protease inhibitor cocktail, phenylmethylsulfonyl fluoride (PMSF), and reduced glutathione (GSH) assay kit were purchased from Sigma-Aldrich (St. Louis, MO). TPA (12-O-tetradecanoylphorbol-13-acetate) was purchased from Cell Signaling Technology while recombinant human interleukin-6 (IL-6) was purchased from PeproTech (Rocky Hill, USA). BAPTA-AM, momelotinib, SP600125, SB203580, and S31-201 were obtained from Selleckchem (Munich, Germany). The TransAM™ STAT3 Transcription Factor Assay Kit was purchased from Active Motif Inc. (Carlsbad CA) while the thioredoxin reductase (TrxR) assay kit was obtained from Abcam (Cambridge, MA). The primary antibodies for cleaved caspases (3 and 9), cleaved PARP, p-STAT3 (Tyr705), STAT3, p-SRC, and SRC were obtained from Cell Signaling Technology (Beverly, MA) while primary antibodies for Bax, Bcl-2, Bcl-xl, Xiap, survivin, ATF4, eIF2*α*, GRP78, GRP98, GAPDH, SHP-1, SHP-2, PTEN, and TrxR1 were obtained from Proteintech (Wuhan, China). The primary antibodies for CHOP, p-JNK, JNK, p-p38, and p38 were obtained from Beyotime Biotechnology. The primary antibodies for p-JAK2 and JAK2 were purchased from Abcam (Cambridge, MA) while primary antibody for p-eIF2*α* was purchased from Santa Cruz Biotechnology (Santa Cruz, CA). Horseradish peroxidase- (HRP-) conjugated secondary antibodies (goat anti-rabbit, goat anti-mouse) were obtained from Sigma-Aldrich.

### 2.2. Cell Culture and Treatments

Human A549 lung adenocarcinoma cells were obtained from American Type Culture Collection (Manassas VA) while NCI-H1650 lung cancer cells and NL-20 normal lung cells were obtained from Shanghai Fuxiang Biotechnology Co. Ltd. Cells were cultured in DMEM supplemented with 10% FBS, 100 units/mL penicillin, and 100 *μ*g/mL streptomycin at 37°C with 5% CO2 in a humidified atmosphere. Cells were treated with PSD-A dissolved in DMSO with final DMSO concentration of 0.5%. DMSO-treated cells were used as control.

### 2.3. Lentivirus Vector Construction and Transduction of A549 Cells

STAT3 (F: 5′-CCGGCTCAGAGGATCCCGGAAATTTCTCGAGAAATTTCCGG G ATCCTCTGAGTTTTTG-3′; R: 5′-AATTCAAAAACTCAGAGGATCCCGGAAATTTCTCGAGAAATTTCCGGGATCCTCTGAG-3′) shRNA oligonucleotides were synthesized, annealed, and inserted into pLKO.1 lentiviral vector (Zhongqiao Xinzhou Biological Technology, Shanghai, China) between AGEI and EcoRI restriction sites. pLKO.1 empty vector was used as nonsilencing control. The above shRNA lentiviral vectors and packaging vectors (Pax2 and Vsvg) were cotransfected into 293FT cells using lipofectamine 2000. 48 hours after transfection, the culture supernatants containing shRNA virus were harvested. A549 cells were then infected with either STAT3 or negative control shRNA virus in the presence of polybrene (8 *μ*g/mL). Puromycin dihydrochloride (1 *μ*g/mL, Sigma-Aldrich) was added to the cells 48 hours postinfection for 12 days to select stable cell lines. STAT3 expression levels were studied by Western blot analysis in the stable cell lines.

### 2.4. Determination of Cell Viability by Trypan Blue Exclusion (TBE) Assay

Lung cancer cells (A549, NCI-H1650) and normal lung cells (NL-20) were cultured in 12-well plates and treated with different concentrations of PSD-A for 24 h. Following treatment, the cells were collected and washed with PBS. The cells were resuspended in 200 *μ*L PBS and incubated with an equal volume of 0.4% trypan blue solution for 5 min at room temperature. The cells were collected by centrifugation, resuspended in PBS, and observed under a microscope. Finally, 100 cells were counted microscopically from three different regions for the percentage of live and dead cells.

### 2.5. Determination of Cell Viability by MTT Assay

The parental and STAT3 knockdown A549 cells were cultured in a 96-well plate for 24 h. The cells were incubated with 10 *μ*L MTT reagent (5 mg/mL) at 37°C for 4 h. Subsequently, 150 *μ*L DMSO was added to dissolve formazan crystals. The absorbance was measured at 570 nm by a microplate reader (Synergy neo HTS multimode microplate reader, BioTek).

### 2.6. Microscopic Observation of Cell Morphology

A549 cells were cultured in 96-well plates for 24 h. The cells were further incubated with different concentrations of PSD-A for 24 h, and cell morphological changes were observed and photographed by a phase contrast microscope (Leica, DMIL LED) equipped with a DFC450C camera.

### 2.7. Colony Forming Assay

Cells were cultured in 6-well culture plates and exposed to different concentrations of PSD-A for 24 h. Following treatment, the cells were washed, trypsinized, and seeded into 6-well plates (500 cells/well) and allowed to grow into colonies for 7 days. The colonies were fixed with 4% paraformaldehyde (PFA) for 10 min, stained with crystal violet solution, and photographed. To quantify the proliferation rate, methanol was added to each well to dissolve crystal violet stain and absorbance was measured at 595 nm.

### 2.8. Apoptosis Assay

A549 cells were cultured in 6-well plates and treated with indicated concentration of PSD-A for 24 h. Following treatment, the cells were harvested, washed with PBS, and resuspended in binding buffer containing 5 *μ*L annexin V and 10 *μ*L PI and incubated in the dark for 15 min according to the kit's instructions. The cells were analyzed by flow cytometry (BD Accuri C6) for the percentage of apoptotic cells.

### 2.9. Evaluation of ROS Generation

Intracellular reactive oxygen species (ROS) generation was measured using 2′,7′-dichlorofluorescein-diacetate (DCFH-DA) probe according to the kit's instructions. Briefly, cells were treated with indicated concentrations of PSD-A in the presence or absence of BAPTA-AM (Ca^++^ chelator) for 24 h. Following treatment, cells were incubated with DCFH-DA for 30 min in the dark. After washing with DMEM for 3 times, DCF fluorescence was measured at an excitation wavelength of 488 nm and an emission wavelength of 525 nm by a fluorescence microplate reader (Synergy neo HTS multimode microplate reader, BioTek).

### 2.10. Measurement of Intracellular Glutathione (GSH)

The intracellular reduced glutathione (GSH) was measured spectrophotometrically using a glutathione-reduced assay kit (Sigma-Aldrich). Briefly, A549 cells were treated with PSD-A with indicated concentrations for 24 h. The cells were collected, and intracellular GSH was measured according to the kit's instructions. The final contents of intracellular GSH were expressed as nmol GSH/mg protein.

### 2.11. Measurement of Thioredoxin Reductase Activity

Thioredoxin reductase (TrxR) activity was measured in cell homogenates using a thioredoxin reductase assay kit according to the manufacturer's instructions (Abcam). Briefly, A549 cells were cultured in 6-well plates and exposed to PSD-A for 24 h. Cells were collected and homogenized on ice by adding 100 *μ*L cold assay buffer. Supernatants were collected by centrifugation at 10,000 ×g for 15 min, and TrxR activity was measured according to the kit's instructions.

### 2.12. Determination of Mitochondrial Membrane Potential (MMP)

MMP was determined by an MMP assay kit (Beyotime Biotechnology) as described by us previously [21]. Briefly, A549 cells were treated with different concentrations of PSD-A, washed with PBS, and stained with JC-1 fluorescent probe for 20 min in the dark. After washing, the fluorescence distribution of JC-1 monomers (green fluorescence) and J-aggregates (red fluorescence) was measured by a fluorescence microplate reader (Synergy neo HTS multimode microplate reader, BioTek). The MMP was calculated by a decrease in red/green fluorescence intensity ratio.

### 2.13. Determination of Intracellular Free Ca^++^

The intracellular free Ca^++^ was detected by fluorescent probe Fluo-3 AM. Being cell membrane permeable, Fluo-3 AM can enter into cells where it is de-esterified into Fluo-3 by the activity of intracellular esterase. The Fluo-3 can specifically combine with Ca^++^ and has a strong fluorescence with an excitation wavelength of 488 nm. The fluorescence intensity of Fluo-3-Ca^++^ complex is proportional to the amount of intracellular free Ca^++^. Briefly, cells were treated with PSD-A in the presence or absence of NAC and BAPTA-AM for 24 h. Following treatments, the cells were collected, washed with PBS, resuspended in serum-free medium containing 5 *μ*M Fluo-3 AM, and incubated in the dark for 30 min with occasional gentle agitation. The cells were washed with PBS and analyzed using a multimode microplate reader (Synergy neo HTS multimode microplate reader, BioTek) at 488 nm excitation and 525 nm emission wavelengths.

### 2.14. STAT3 DNA Binding Assay

A549 cells were incubated with or without PSD-A for 4 h and then further incubated with IL-6 for 1 h. The nuclear extracts were collected, and binding of STAT3 to DNA was measured with an ELISA-based TransAM STAT3 assay kit according to the manufacturer's instructions (Active motif).

### 2.15. Analysis of Caspase-4 Activity

A549 cells were exposed to different concentrations of PSD-A for 24 h. The cell lysates were prepared, and protein concentration was measured by BCA kit. The activity of caspase-4 was then measured according to the kit's instructions (Beyotime Biotechnology) An equal amount of cell extracts (30 *μ*g) was added into a 96-well plate and incubated with Ac-LEVD-*p*NA (Beyotime Biotechnology) for 2 h at 37°C for cleavage of Ac-LEVD-*p*NA to *p*-NA. The caspase-4 activity was then determined by measuring the absorbance of *p*-NA at 405 nm. The relative caspase-4 activity was measured as a ratio of emission of treated cells to untreated cells.

### 2.16. Western Blotting

After drug treatment, adherent and floating cells were collected and washed with cold PBS and whole cell lysates were prepared using cell lysis buffer containing 20 mM Tris (pH 7.5), 150 mM NaCl, 1% Triton X-100, 50 mM NaF, 0.1 mM PMSF, sodium pyrophosphate, *β*-glycerophosphate, EDTA, Na_3_VO_4_, and leupeptin (Beyotime Biotechnology) on ice for 30 min. The protein concentration was determined by an enhanced BCA protein assay kit (Beyotime Biotechnology). A total of 40 *μ*g protein was separated on 10% sodium dodecyl sulfate polyacrylamide gel electrophoresis (SDS-PAGE) and transferred to polyvinylidene difluoride (PVDF) membrane. After blocking with 5% skim milk, the membranes were incubated with respective primary antibodies overnight at 4°C. After washing with Tris-buffered saline-Tween (TBST) solution, the membranes were incubated with HRP-conjugated goat anti-rabbit IgG or goat anti-mouse IgG secondary antibodies for 1 h at room temperature. After washing with TBST 3 times, the immunoreactive bands were detected by Immobilon Western chemiluminescent HRP substrate (Millipore, Billerica, MA) and chemiluminescence images were obtained using MicroChemi 4.2 imaging system (DNR Bio-Imaging system). All the blots shown are representative of three separate experiments. Quantification of protein bands was performed using ImageJ software. GAPDH was used as loading control and detected in every individual blot. The values above the figures represent the relative density of the bands normalized to GAPDH from three repeated experiments.

### 2.17. Molecular Docking

The three-dimensional structure of STAT3 (PDB code: 1BG1) was obtained from the PDB database (Protein Data Bank; http://www.rcsb.org/), and the proscillaridin A structure was adopted from the PubChem Compound database (https://www.ncbi.nlm.nih.gov/pccompound/). Before docking simulations, the crystallographic water molecules were removed and the missing hydrogen atoms were added onto STAT3 crystal structure. STAT3 monomer was added with pol-hydrogen, with given Kullman-Uni charge. The program LIGPLOT [22] and PyMOL (v 1.3) were used to visualize the interactions of docked protein-ligand complexes. The AutoDock Tools (ADT) was used to transform the Mol2 format ligand and the pdb format receptor into the pdbqt format which could be used by AutoDock 4.2. The docking gridbox of the receptor was also set by the ADT, whose number of grid points in XYZ is 126 × 126 × 126 and spacing is 0.375 Å. The Lamarckian genetic algorithm of AutoDock 4.2 was exploited for docking simulations, and 100 runs were performed [23].

### 2.18. Statistical Analysis

The results are expressed as mean ± standard error mean (SEM) of 3 different experiments and statistically compared with the control group or within the groups using one-way ANOVA for more than two data sets followed by Tukey's multiple comparison test or two-way ANOVA to analyze data with more than two variables. Student's *t*-test was used to determine the significance when only two groups were compared, and *P* < 0.05 was considered statistically significant.

## 3. Results

### 3.1. PSD-A Induces Cytotoxic Effects in Lung Cancer Cells

The cytotoxic effects of PSD-A on NSCLC were evaluated using A549 and NCI-H1650 cells by the trypan blue exclusion (TBE) method. PSD-A increased the number of dead cells in a dose-dependent manner and was found to be equally toxic to both A549 and H1650 cells (Figure 1(a)). Apart from cancer cells, the cytotoxicity of PSD-A was also assessed in NL-20 normal lung cells. PSD-A exhibited less cytotoxic effects to NL-20 cells at lower concentrations (10–50 nM); however, at higher concentrations (100–200 nM), its cytotoxic effect was comparable to cancer cells (Figure 1(a)). Therefore, 25 and 50 nM concentrations of PSD-A were selected for further mechanistic study and A549 cell line was selected as a model cell line for this study. To further examine the effect of PSD-A on cell morphology, we exposed A549 cells to PSD-A in a dose-dependent manner for 24 h and observed morphological changes under a light microscope. As shown in Figure 1(b), control cells exhibited an adhesive and widened morphology while cells exposed to PSD-A showed severe morphological alterations characteristically associated with cell death such as rounded shape, loss of anchorage, presence of floating cells in the medium, and reduction in the total number of cells compared to that of normal control cells. The growth inhibitory effect of PSD-A on A549 cell growth was evaluated by a clonogenic assay. As shown in Figures 1(c) and 1(d), PSD-A treatment remarkably suppressed colony formation in A549 cells in a dose-dependent manner.

### 3.2. PSD-A Induces Apoptotic Cell Death in A549 Cells

**Figure 2:**
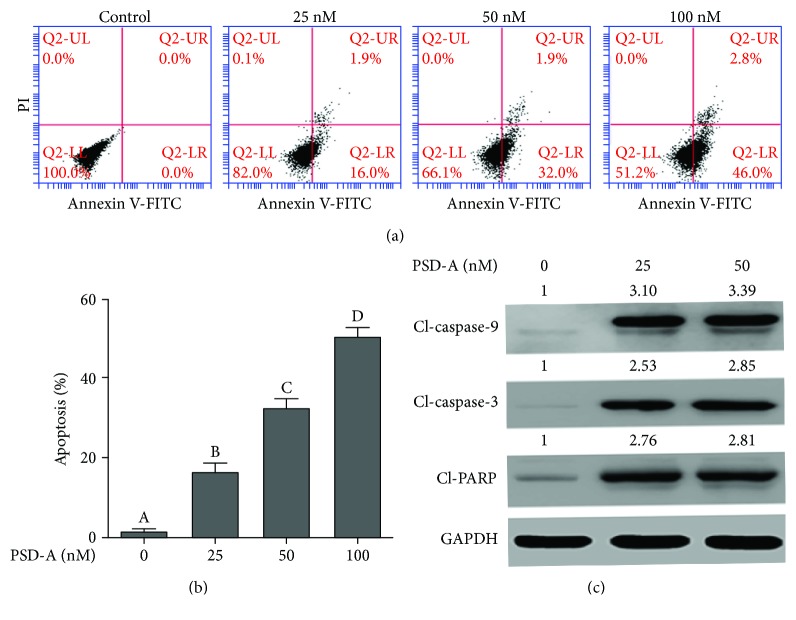
Effect of PSD-A on cell apoptosis. (a, b) A549 cells were treated with PSD-A in a dose-dependent manner for 24 h. Cells were stained with annexin V-FITC/PI, and samples were analyzed by flow cytometry for the detection of apoptosis. Data are expressed as mean ± SEM (*n* = 3). Columns not sharing the same superscript letters differ significantly (*P* < 0.05). (c) Cells were incubated with indicated concentrations of PSD-A for 24 h. Extracts were prepared and subjected to Western blot for the expressions of apoptosis markers (cleaved caspase-9, caspase-3, and PARP).

The data from TBE assay showed that PSD-A induces cell death in A549 cells. To ascertain the PSD-A-mediated cell death and to study the nature of cell death, we performed flow cytometry analysis of apoptosis by staining the cells with annexin V-FITC and PI. As shown in Figures 2(a) and 2(b), PSD-A treatment significantly increased annexin V-positive cells (early apoptosis) in a dose-dependent manner. A small population of cells (about 2%) positive to both annexin V and PI (late apoptosis/necrosis) has been observed in all treatment groups while that of cells positive to only PI (necrotic cells) has not been detected in any treatment group indicating apoptotic nature of cell death. Cleavage of caspases and poly (ADP ribose) polymerase (PARP) are the hallmarks of apoptotic cell death [24]. To further confirm apoptotic cell death, we measured the expressions of cleaved caspase-9, caspase-3, and PARP. As shown in Figure 2(c), PSD-A treatment remarkably increased the expression of cleaved caspase-9, caspase-3, and PARP. Collective data indicate clearly that PSD-A induces apoptosis in A549 lung cancer cells.

### 3.3. PSD-A Induces Oxidative Stress in A549 Cells

**Figure 3:**
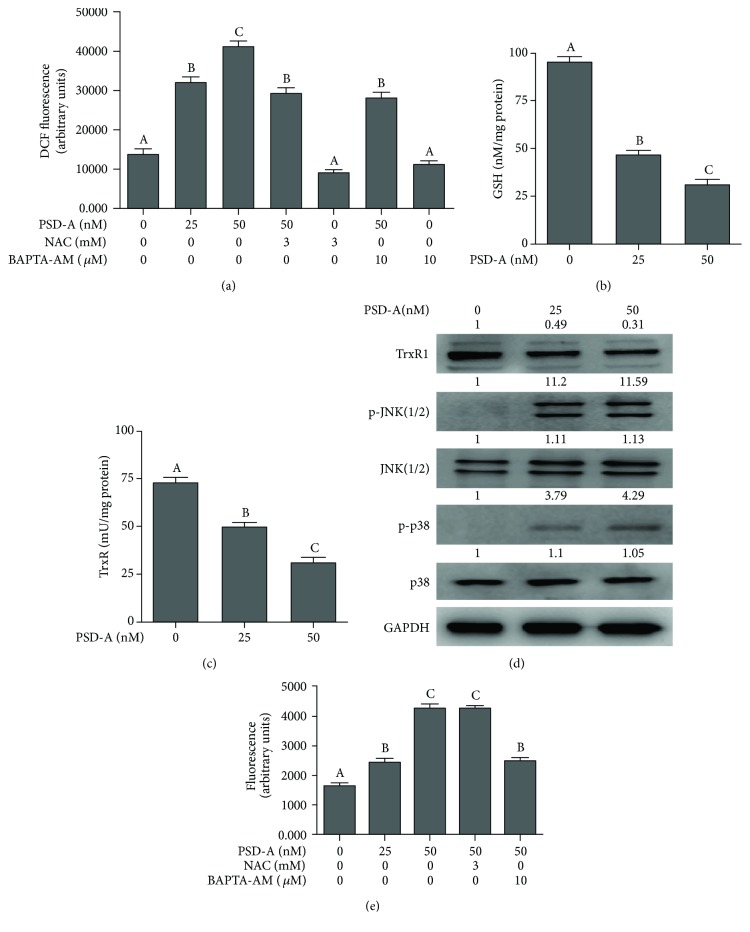
PSD-A stimulates oxidative stress in A549 cells. (a) A549 cells were treated with PSD-A in the presence or absence of BAPTA-AM (10 *μ*M) and NAC (3 mM) for 24 h, and ROS generation was determined by staining the cells with DCFH-DA. (b, c) A549 cells were treated with PSD-A for 24 h and intracellular level of GSH, and TrxR activity was measured according to the kit's instructions. Data in (a–c) are expressed as mean ± SEM (*n* = 3). Columns not sharing the same superscript letters differ significantly (*P* < 0.05). (d) Cells were treated with PSD-A for 24 h, and expressions of TrxR1, p-JNK1/2, JNK1/2, p-p38, and p38 were measured by Western blot. (e) Cells were treated with PSD-A for 24 h as indicated, and intracellular free Ca^++^ was measured using Fluo-3 AM fluorescent probe as described in Materials and Methods. Data are expressed as mean ± SEM (*n* = 3). Columns not sharing the same superscript letters differ significantly (*P* < 0.05).

Under normal physiological conditions, cellular redox homoeostasis is maintained by a precise balance between reactive oxygen species (ROS) production and antioxidant system's capacity to scavenge ROS. Intracellular reduced glutathione (GSH) and thioredoxin (Trx) are two major antioxidant systems of the cells which play an important role in maintaining cellular redox status by trapping ROS or by reversing the formation of disulfide oxidation products. An imbalance between ROS production and antioxidant system's ability to neutralize ROS is termed oxidative stress [24]. Many chemotherapeutic drugs kill cancer cells by disturbing the redox homoeostasis of cells [25, 26]. We were interested to know if PSD-A could induce oxidative stress in A549 cells. Therefore, we measured the level of intracellular ROS in control and PSD-A treated cells. The data showed that PSD-A treatment for 24 h increased the level of ROS in a dose-dependent manner. This PSD-A-induced ROS production was partially inhibited by pretreatment of cells with BAPTA-AM (10 *μ*M) and NAC (3 mM) (Figure 3(a)). The data indicate that PSD-A induces ROS generation by increasing intracellular Ca^++^ level. Next, we wonder if PSD-A could also inhibit the antioxidant system of the cells. Therefore, we measured the intracellular GSH level and thioredoxin reductase (TrxR) activity in control and drug-treated cells. The data demonstrated that PSD-A increased GSH depletion and inhibited TrxR activity in a dose-dependent manner (Figures 3(b) and 3(c)). In addition to TrxR activity, PSD-A also decreased the expression of TrxR1 as shown in Figure 3(d). Collective data from above experiments indicate clearly that PSD-A induces oxidative stress in A549 cells after 24 h drug treatment.

### 3.4. PSD-A Induces p38 and JNK Activation

The mammalian mitogen-activated protein kinase (MAPK) family consists of extracellular signal-regulated kinase (ERK), p38, and c-Jun N-terminal kinase (JNK) which are activated by diverse extracellular and intracellular stimuli including growth factors, cytokines, hormones, and various cellular stressors such as oxidative stress and endoplasmic reticulum stress. The JNK and p38 proteins are activated in response to cellular stresses and play an important role in cell apoptosis [27]. Therefore, we measured the expression of p-JNK/JNK and p-p38/p38. The data showed that PSD-A increased phosphorylation of JNK and p38 without affecting the expression of total JNK and p38 (Figure 3(d)).

### 3.5. PSD-A Increases Intracellular Ca^++^ in A549 Cells

Cardiac glycosides have been shown to increase intracellular Ca^++^ level due to their inhibitory effect on Na^+^/K^+^-ATPase pump [17, 18]. Therefore, we measured intracellular Ca^++^ level in A549 cells in response to PSD-A treatment. The data demonstrated that PSD-A significantly increased intracellular free Ca^++^ in A549 cells in a dose-dependent manner (Figure 3(e)). To test the possibility of ROS generation in intracellular Ca^++^ release, we determined the effect of PSD-A on Ca^++^ release in the presence of NAC, a ROS scavenger. As shown in Figure 3(e), pretreatment of cells with NAC failed to inhibit intracellular Ca^++^ release in A549 cells. However, BAPTA-AM, a Ca^++^ chelator, effectively decreased PSD-A-mediated increase in intracellular Ca^++^ level.

### 3.6. PSD-A Induces Mitochondrial Apoptosis in A549 Cells

**Figure 4:**
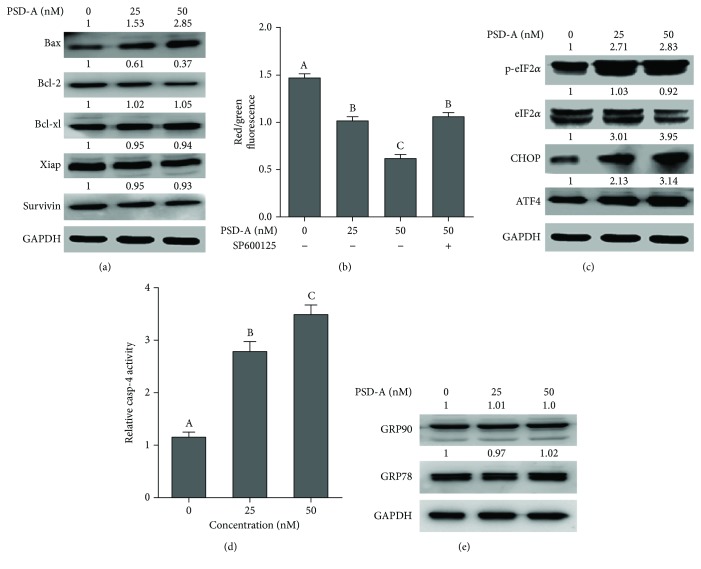
PSD-A induces mitochondrial dysfunctions and ER stress in A549 cells. (a) Cells were treated with indicated concentrations of PSD-A for 24 h. Cell lysates were prepared and subjected to Western blot for the expressions of Bax, Bcl-2, Bcl-xl, Xiap, and survivin. GAPDH was used as loading control. (b) Cells were treated with PSD-A for 24 h in the presence or absence of BAPTA-AM (10 *μ*M), and MMP was determined by staining the cells with JC-1 according to the product's instructions. Data are expressed as mean ± SEM (*n* = 3). Columns not sharing the same superscript letters differ significantly (*P* < 0.05). (c, d) Cells were exposed to the indicated concentrations of PSD-A for 24 h. Total cell lysates were extracted, and expressions of p-eIF2*α*, eIF2*α*, CHOP, ATF4, GRP90, and GRP78 were measured by Western blot analyses. (e) Cells were treated with PSD-A for 24 h, and activity of caspase-4 was measured according to the kit's instructions. PSD-A increased the activity of caspase-4. Data are expressed as mean ± SEM (*n* = 3). Columns not sharing the same superscript letters differ significantly (*P* < 0.05).

Bcl-2 family protein modulation and mitochondrial membrane potential (MMP) dissipation are the hallmarks of mitochondrial apoptosis [28]. To gain better insight into PSD-A-mediated apoptosis, we measured the expressions of Bcl-2 family proteins. The data demonstrated that PSD-A increased the expression of proapoptotic bax and decreased the expression of antiapoptotic bcl-2 proteins without affecting the expression of bcl-xl (Figure 4(a)). We also measured the expression of inhibitors of apoptosis xiap and survivin. As shown in Figure 4(a), PSD-A did not modulate the expressions of xiap and survivin. Next, we determined MMP in control and drug-treated cells. As evident from Figure 4(b), PSD-A significantly reduces MMP in A549 cells. Pretreatment of cells with SP600125 (JNK inhibitor) improved MMP of PSD-A-treated A549 cells. The data indicate that PSD-A-mediated apoptosis is associated with mitochondrial dysfunction.

### 3.7. PSD-A Induces ER Stress in A549 Cells

Previous studies have shown that the MAPK signaling pathway is involved in various cellular mechanisms such as apoptosis and ER stress [29]. Therefore, we measured the expressions of various proteins involved in ER stress response by Western blot. The data showed that the dose-dependent treatment of PSD-A remarkably increased the expression of phosphorylated eukaryotic translation initiation factor 2*α* (p-eIF2*α*) and activating transcription factor 4 (ATF4) in cells. Increased expression of ATF4 has been documented to induce the expression of the prodeath transcriptional regulator, C/EBP homologous protein (CHOP), also known as growth arrest, and DNA damage-inducible gene 153 (GADD153). Induction of CHOP expression and caspase-4 activation are the hallmarks in the commitment of ER stress-induced apoptosis [30]. Therefore, we measured the expression of CHOP and caspase-4 activation. As shown in Figures 4(c) and 4(d), PSD-A enhanced the expression of CHOP and induced caspase-4 activation in A549 cells in a dose-dependent manner. On the other hand, PSD-A did not change the expressions of cytoprotective ER chaperones, glucose-regulated protein 78 (GRP78), and GRP90 (Figure 4(e)) indicating that the CHOP-dependent prodeath pathway is dominant over the survival pathway in A549 cells exposed to PSD-A.

### 3.8. SP600125 Partially Inhibits PSD-A-Induced Apoptosis and ER Stress in A549 Cells

**Figure 5:**
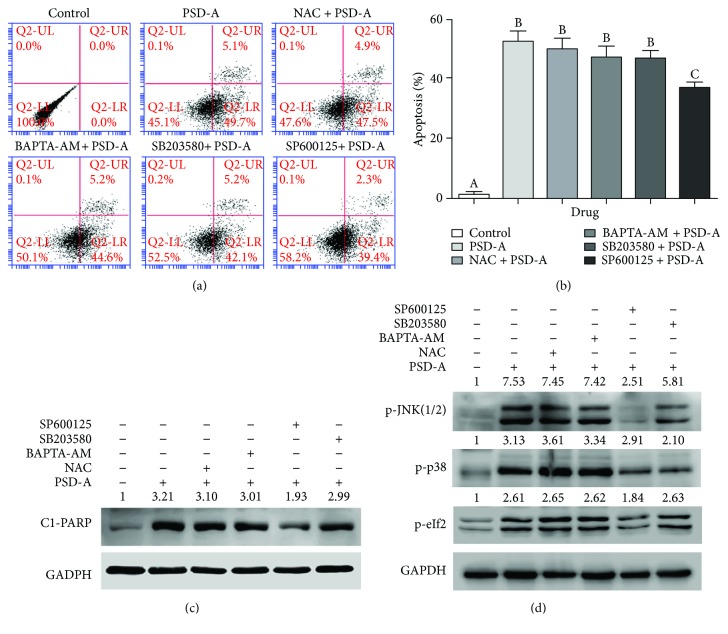
PSD-A induces apoptosis and ER stress via JNK activation. (a) A549 cells were treated with 100 nM PSD-A in the presence or absence of NAC (3 mM), BAPTA-AM (10 *μ*M), SB203580 (5 *μ*M), and SP600125 (20 *μ*M) for 24 h, and apoptosis was determined using flow cytometry. (b) Data from (a) are expressed as mean ± SEM (*n* = 3). Columns not sharing the same superscript letters differ significantly (*P* < 0.05). (c) A549 cells were treated with PSD-A in a similar fashion as described above; total cell lysates were extracted and subjected to Western blot for the expression of cleaved PARP. (d) A549 cells were treated with 50 nM PSD-A in the presence or absence of various inhibitors as indicated for 8 h. The expressions of p-JNK(1/2), p-p38, and p-eIF2*α* were measured by Western blot.

To further explore the in-depth molecular mechanism and to evaluate the functional role of various cellular events in PSD-A-induced apoptosis, we treated A549 cells with 100 nM PSD-A for 24 h in the presence or absence of various inhibitors and performed flow cytometry analysis of apoptosis. NAC (ROS inhibitor), BAPTA-AM (Ca^++^ chelator), and SB203580 (p38 MAPK inhibitor) failed to inhibit PSD-A-induced apoptosis in A549 cells. However, SP600125, a broad spectrum JNK inhibitor, partially protected the cells from PSD-A-induced apoptosis (Figures 5(a) and 5(b)). To further validate these results, we treated the cells with PSD-A in similar conditions and measured the expression of cleaved PARP in total cell lysates by Western blot. In line with data obtained from the flow cytometry analysis of apoptosis, SP600125 reduced the expression of cleaved PARP in PSD-A-treated cells while NAC, BAPTA-AM, and SB203580 failed to inhibit PSD-A-induced expression of cleaved PARP (Figure 5(c)). The data indicate that JNK activation plays an important role in PSD-A-induced apoptosis in A549 cells.

To further exclude the possible role of intracellular ROS generation and Ca^++^ release in activation of MAPK signaling and ER stress, we exposed the cells to PSD-A in the presence or absence of NAC, BAPTA-AM, SB203580, and SP600125 for 8 h and measured the expression of p-JNK, p-p38, and p-eIF2*α* in cellular extracts by Western blot. As shown in Figure 5(d), PSD-A increased the phosphorylation of JNK (1/2), p38, and eIF2*α* at 8 h time point. SP600125 partially inhibited the phosphorylation of eIF2*α* indicating that PSD-A induces ER stress in A549 cells at least in part via JNK activation. On the other, no effect of NAC, BAPTA-AM, and SB203580 has been observed on phosphorylation of eIF2*α* in PSD-A-treated cells. Similarly, NAC and BAPTA-AM did not inhibit PSD-A-mediated phosphorylation of JNK and p38. Collective data indicate that PSD-A induces apoptosis, MAPK signaling, and ER stress without the involvement of ROS generation and intracellular Ca^++^ release in A549 cells while JNK activation plays an important role in PSD-A-induced ER stress and apoptosis.

### 3.9. PSD-A Inhibits Constitutive and Inducible STAT3 Activation in A549 Cells

**Figure 6:**
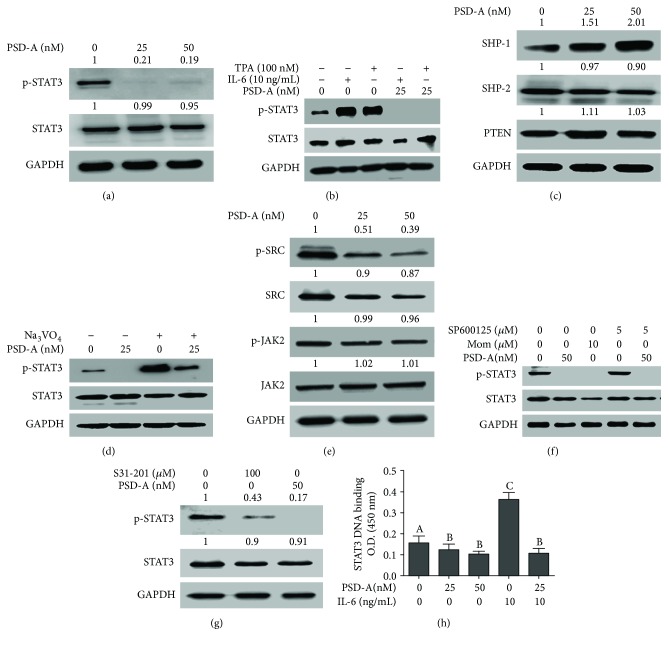
PSD-A inhibits STAT3 signaling pathway in A549 cells. (a) PSD-A inhibits constitutive STAT3 activation at tyrosine 705. Cells were treated with PSD-A for 24 h, and expression of p-STAT3 and total STAT3 was measured using Western blot. (b) PSD-A inhibits inducible STAT3 activation in A549 cells. A549 cells were pretreated with PSD-A for 4 h and then stimulated with 100 nM TPA and 10 ng/mL IL-6 for 1 h. Extracts were prepared and subjected to Western blot for the expression of p-STAT3 and STAT3. (c) Cells were treated with PSD-A for 24 h, and expressions of phosphatases (STAT3-negative regulators) were determined by Western blot. PSD-A increased the expression of SHP-1 without affecting SHP-2 and PTEN. (d) Cells were treated with 25 nM PSD-A in the presence or absence of Na_3_VO_4_ (100 *μ*M) for 24 h. Cell lysates were collected and subjected to Western blot for the expression of p-STAT3 and STAT3. Na_3_VO_4_ pretreatment reversed the suppressive effect of PSD-A on STAT3 indicating that tyrosine phosphatases play an important role in PSD-A-mediated STAT3 inhibition. (e) Cells were treated with PSD-A for 4 h, and expressions of tyrosine kinases (p-SRC/SRC and p-JAK2/JAK2) were measured by Western blot. PSD-A suppressed the phosphorylation of SRC but did not affect p-JAK2 expression. (f) Cells were treated with or without PSD-A in the presence or absence of momelotininb (JAK inhibitor) and SP600125 (JNK inhibitor) for 4 h. Proteins were extracted, and expression of p-STAT3 and STAT3 was detected by Western blot. (g) Cells were treated with PSD-A (50 nM) and S31–201 (100 *μ*M) for 4 h, and expression of p-STAT3 was measured in cell lysates by Western blot. (h) A549 cells were incubated with or without PSD-A for 4 h and then further incubated with IL-6 for 1 h. The nuclear extracts were then collected and assayed for STAT3 DNA-binding activity according to the instructions of the kit. Columns not sharing the same superscript letters within the groups differ significantly (*P* < 0.05).

STAT3 is persistently activated by phosphorylation at tyrosine 705 in NSCLC and has been shown to play a vital role in cell survival and drug resistance. Therefore, we investigated the effect of PSD-A on STAT3 activation. We found that PSD-A treatment for 24 h effectively suppressed STAT3 phosphorylation at tyrosine 705 in A549 cells (Figure 6(a)). Next, we evaluated the effect of PSD-A on inducible STAT3 activation. For this, we induced STAT3 activation using IL-6 and TPA. As shown in Figure 6(b), IL-6 (10 ng/mL) and TPA (100 nM) treatment for 1 h resulted in STAT3 activation while the pretreatment of cells with 25 nM PSD-A for 4 h effectively abrogated the IL-6- and TPA-induced STAT3 activation.

### 3.10. Sodium Orthovanadate Reverses PSD-A-Induced Inhibition of STAT3 Activation

Since the activation of STAT3 is negatively regulated by protein tyrosine phosphatases (PTPs), we measured the expression of PTPs implicated in regulating STAT3 activation. As shown in Figure 6(c), PSD-A increased the expression of SHP-1 while expressions of SHP-2 and PTEN remain unaffected. To probe the functional role of PTPs in PSD-A-induced inhibition of STAT3 activation, we treated the cells with PSD-A in the presence of sodium orthovanadate (100 *μ*M), a broad spectrum tyrosine phosphatase inhibitor. The data demonstrated that pretreatment with sodium orthovanadate reversed the PSD-A-mediated suppression of STAT3 activation (Figure 6(d)). These results suggest that PTP activation plays an important role in PSD-A-induced inhibition of STAT3 activation in A549 cells.

### 3.11. Effect of PSD-A on STAT3 Upstream Tyrosine Kinases

STAT3 is activated by various upstream signaling molecules including Janus-activated kinases (JAKs), Src family kinases, and MAPKs [12]. To investigate whether the inhibitory effect of PSD-A on STAT3 activation is due to the suppression of upstream tyrosine kinases, we measured the expressions of p-JAK2, JAK2, p-Src, and Src in PSD-A-treated and control cells. As shown in Figure 6(e), PSD-A suppressed the phosphorylation of Src while it did not decrease the phosphorylation and expression of JAK2. Conversely, momelotinib (JAK inhibitor) effectively inhibited STAT3 activation in A549 cells. The data suggest that although PSD-A-induced inhibition of STAT3 activation seems independent of JAK-2, yet JAK-2 inhibition plays an important role in STAT3 inhibition in A549 cells. As PSD-A increased the phosphorylation of JNK, we were interested to know if the inhibition of STAT3 activation by PSD-A is associated with JNK activation. To answer this question, we treated the cells with SP600125 (JNK inhibitor) in the presence or absence of PSD-A and measured the phosphorylation of STAT3. The data demonstrated that SP600125 neither increased the phosphorylation of STAT3 compared to control cells nor abrogated PSD-A-mediated inhibition of STAT3 phosphorylation (Figure 6(f)), indicating that PSD-A inhibits STAT3 activation through a mechanism that does not involve JNK activation.

Next, we compared the inhibitory effect of PSD-A and S31-201, (a commercially available STAT3 inhibitor) on STAT3 phosphorylation to evaluate the efficacy of PSD-A as a potent STAT3 inhibitor. The data showed that PSD-A (50 nM) has a greater inhibitory effect on STAT3 activation compared to S31-201 (100 *μ*M) despite the low PSD-A concentration used (Figure 6(g)). Finally, we determined STAT3 DNA-binding activity.  Figure 6(h) shows that consistent with the inhibition of STAT3 activation, PSD-A decreased STAT3 DNA binding in a dose-dependent manner.

### 3.12. PSD-A Was Predicted to Bind to SH2 Domain of STAT3 by Computational Docking

**Figure 7:**
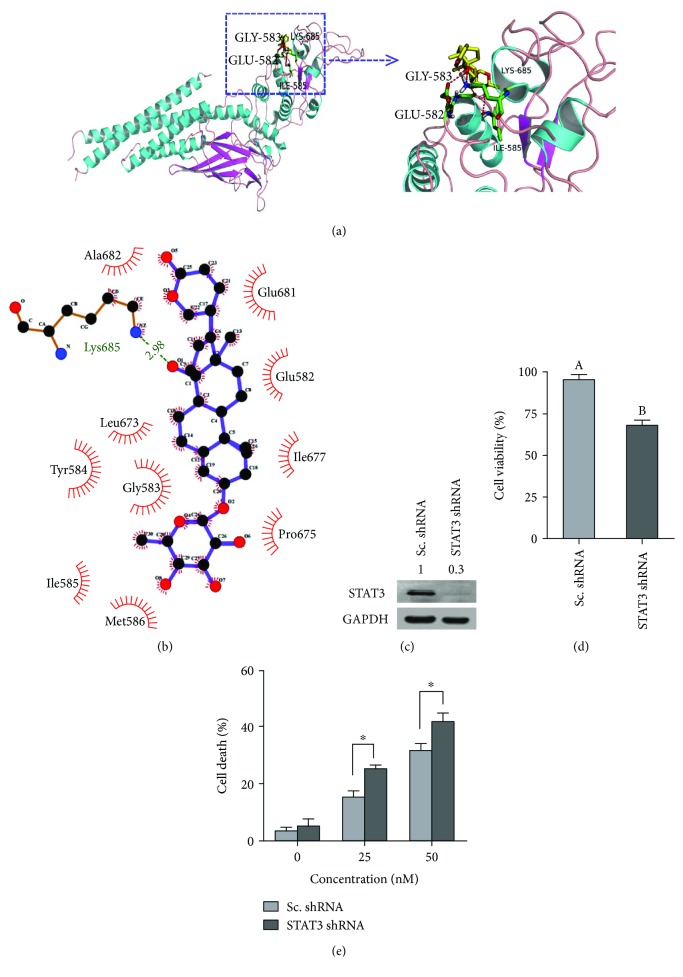
Computational modeling of PSD-A binding with STAT3 and effect of STAT3 knockdown on cell growth and death. (a) The docking simulation results obtained from PyMOL showing hydrogen bonding between PSD-A and STAT3 SH2 domain. (b) Two-dimensional representation of these interactions between PSD-A and STAT3, plotted by LIGPLOT. Hydrogen bond interactions are indicated as green dotted lines. (c) Western blot expression of STAT3 in scrambled (Sc) treated and STAT3 knockdown A549 cells. (d) Evaluation of the effect of STAT3 knockdown on proliferation of A549 cells by MTT assay. (e) Effect of STAT3 knockdown on PSD-A-induced cell death in A549 cells was determined by TBE assay. Data are expressed as mean ± SEM of 3 independent experiments. Statistical significance between scrambled shRNA and STAT3 shRNA was determined by two-way ANOVA followed by the Bonferroni post hoc test for multiple comparisons. ^∗^*P* < 0.05.

The docking simulation results revealed that the GLU582, GLY583, ILE585, and LYS685 residues form hydrogen bonds with PSD-A (Figure 7(a)). This indicates that PSD-A mainly interacts with the SH2 domain of STAT3. Two-dimensional representation of these interactions, plotted by LIGPLOT, is shown in Figure 7(b). The results from LIGPLOT indicate that PSD-A is predicted to form stable hydrogen bonds with the amino group of LYS685. In addition to hydrogen bonding, the hydrophobic bonding interactions of amino acid residues Glu582, Gly583, Tyr584, Ile585, Met586, Leu673, Pro675, Ile677, Glu681, and Ala682 were observed at the perimeter of PSD-A. Not exactly the same results were obtained from PyMOL and LIGPLOT because they adopted different criteria.

### 3.13. STAT3 Knockdown Inhibits Proliferation and Augments PSD-A-Induced Toxicity

In order to characterize the functional role of STAT3 activation in A549 cells' proliferation and apoptosis, we generated a stable STAT3 knockdown A549 cell line. As shown in Figure 7(c), STAT3 protein expression was effectively eliminated from A549 cells after STAT3 shRNA transduction. Next, we examined the effect of STAT3 knockdown on cell proliferation by MTT assay. The data demonstrated that STAT3 knockdown significantly inhibited the proliferation of A549 cells (Figure 7(d)). To further confirm the role of STAT3 knockdown in PSD-A-mediated cell death, we measured cell death by TBE assay. As shown in Figure 7(e), STAT3 knockdown significantly increased PSD-A-induced cell death in A549 cells.

## 4. Discussion

While cardiac glycosides are being used clinically for the treatment of cardiac arrest effectively, in the present study, we have investigated the anticancer mechanism of PSD-A, a bufadienolide cardiac glycoside in A549 lung adenocarcinoma cells. The study demonstrated that PSD-A could effectively kill NSCLC cells at extremely low concentration (25–50 nM) by interacting with various novel cellular targets and upstream mechanisms. The major mechanisms identified for the anticancer activity of PSD-A in the present study were JNK activation, mitochondrial dysfunction, ER stress, and inhibition of the STAT3 signaling pathway which ultimately contributed for the induction of apoptosis.

Firstly, we showed that PSD-A exerts cytocidal effects in A549 and H1650 NSCLC cells while exhibiting a less-toxic effect on NL-20 normal lung cells at a concentration range of 25–50 nM. However, at higher concentrations (100–200 nM), the cytotoxicity of PSD-A on NL-20 normal lung cells was comparable to A549 and H1650 lung cancer cells indicating that PSD-A is nonselective at higher concentrations in our study model. Further study is needed to investigate the selectivity index of PSD-A on a large panel of cancer and normal cell lines for its therapeutic potential. Moreover, medicinal chemistry studies may be needed to improve the selectivity index of PSD-A. In the present study, we selected 25 and 50 nM concentrations to demonstrate the anticancer mechanism of PSD-A using *in vitro* cell studies. PSD-A induced several morphological changes in A549 cells characteristically associated with cell death and inhibited growth in an irreversible fashion as depicted from clonogenic assay. Cardiac glycosides have been reported to induce apoptotic as well as autophagic cell death in various cancer cells [17]. In the present study, PSD-A has been shown to induce apoptotic cell death in A549 lung cancer cells as evidenced by annexin V-FITC binding, caspases activation, and PARP cleavage which are considered the hallmarks of apoptotic cell death [31].

It is well established now that cardiac glycosides mediate cardiotonic effects by inhibiting Na^+^/K^+^-ATPase pump. Inhibition of Na^+^/K^+^-ATPase pump by cardiac glycosides results in an increase in intracellular Ca^++^ level which increases contractility of the heart [17, 18]. Other studies reported that an increase in intracellular Ca^++^ level leads to induction of ROS generation and apoptosis [32, 33]. Although the ability of cardiac glycosides to increase intracellular Ca^++^ level by inhibiting Na^+^/K^+^-ATPase pump has initially been suggested the primary event for their anticancer activity, however, this hypothesis has not always been verified. In fact, cardiac glycosides have also been shown to induce cell death without modulating the intracellular Ca^++^ concentration in glioblastoma and prostate cancer cells [34, 35]. The current study was designed to understand the role of various signaling molecules in PSD-A-mediated apoptosis in A549 lung cancer cells. In order to get into better insight, we measured the effect of PSD-A on intracellular free Ca^++^, ROS generation, GSH, and thioredoxin. PSD-A treatment for 24 h significantly increased intracellular free Ca^++^, ROS generation, and decreased GSH and inhibited TrxR activity and expression. Although NAC and BAPTA-AM significantly inhibited PSD-A-induced ROS generation, however, both inhibitors failed to prevent apoptosis induced by PSD-A. The findings demonstrate that ROS generation by PSD-A is not a triggering signal for apoptosis in our study model but rather a consequence of cell death. We have also excluded the possibility that PSD-A induces apoptosis by increasing intracellular free Ca^++^ since BAPTA-AM did not inhibit apoptosis.

Apoptosis is a highly synchronized mode of cell death. Activation of apoptosis in cancer cells by natural compounds has been suggested one of the most important strategies to set the cancer cells on the road to ruin [11]. Many anticancer drugs induce apoptosis in cancer cells through the death receptor pathway, the mitochondrial pathway, or the ER stress-mediated pathway [36]. Mitochondrial apoptosis is regulated by Bcl-2 family proteins. Under normal physiological conditions, bax, a proapoptotic protein, resides in the cytosol and is negatively regulated by the antiapoptotic protein bcl-2. Thus, the bcl-2/bax ratio determines the fate of the cell. In the presence of apoptotic stimulus, the bcl-2/bax ratio decreases which results in mitochondrial membrane potential disruption. Bcl-2 family protein modulations and MMP dissipation are the hallmarks of mitochondrial apoptosis [28, 37]. In line with the established parameters of mitochondrial apoptosis, an increase in bax expression while a decrease in bcl-2 expression followed by MMP dissipation was observed in PSD-A-treated cells.

The role of ER stress in the pathogenesis of various diseases such as neurodegeneration, inflammation, and cancer is well established. A large body of literature evidence indicates that ER stress is accompanied by oxidative stress, mitochondrial dysfunction, and apoptosis and plays a vital role in drug-induced toxicity [29]. The ER stress is counteracted by an adaptive program called unfolded protein response (UPR). The UPR primarily acts as a prosurvival pathway by upregulating the expressions of ER chaperones GRP-78/Bip and GRP-90 which increase the protein folding capacity as well as target misfolded protein for degradation and inhibits translation by inducing phosphorylation of eIF2*α*. Although the primary function of UPR is to promote cell survival by restoring ER homoeostasis, however, it switches to initiate the process of cell death if ER stress persists or overwhelms [38]. In the present study, PSD-A induces ER stress as indicated by increased phosphorylation of eIF2*α*, the key indicator of ER stress [39]. While phosphorylation of eIF2*α* inhibits the translation of proteins, mRNA of ATF4 is preferentially translated into protein under such conditions. ATF4 promotes the expressions of cytoprotective ER chaperones to promote cell survival as well as proapoptotic CHOP depending upon the UPR conditions [40]. The fate of UPR as prosurvival or proapoptotic is determined by the dominant expression of one pathway over the other. Here, in this study, PSD-A increased the expression of CHOP and promoted the activity of caspases-4 without elevating the expression of cytoprotective ER chaperones, GRP-78, and GRP-90 indicating that the CHOP-dependent proapoptotic ER stress pathway is dominant over the survival pathway in PSD-A treated A549 cells.

The MAPK signaling cascade consists of p38, JNK, and ERK (1/2) and is involved in various cellular processes such as cell proliferation, survival, and death. JNK and p38 activation mediates signal transduction leading to cell death while ERK (1/2) activation promotes cell survival [29, 41]. Here, we found that PSD-A increased phosphorylation of p38 and JNK in A549 cells after 24 h treatment. Previous reports indicate that several key mechanisms associated with drug-induced toxicity such as mitochondrial dysfunction, ER stress, autophagy, and apoptosis are regulated by MAPK signaling pathways [29, 42, 43]. Here, in this study, using a MAPK inhibition approach by pharmacological inhibitors, we investigated the possible functional role of JNK and p38 in PSD-A-induced ER stress and apoptosis. Both SB203580 (p38 inhibitor) and SP600125 (JNK inhibitor) effectively inhibited the activation of p38 and JNK; however, only SP600125 inhibited PSD-A-induced apoptosis as evident from the flow cytometry analysis of apoptosis and PARP cleavage. Next, we asked if PSD-A-mediated ER stress and mitochondrial dysfunction are regulated by p38 and JNK activation. Only SP600125 inhibited PSD-A-mediated phosphorylation of eIF2*α* and improved mitochondrial membrane potential. It is worth noting that pharmacological inhibition of JNK only partially protected the cells from PSD-A-induced apoptosis suggesting the involvement of additional mechanisms in PSD-A-induced apoptosis. Although the role of JNK activation in PSD-A-induced ER stress and apoptosis is defined in the current study, however, the functional role of ER stress in the induction of apoptosis in response to PSD-A treatment remains unknown.

STAT3 is frequently found activated in several cancers including EGFR wild type as well as EGFR-mutated NSCLC [44]. STAT3 activation has been found to be associated with cancer progression including survival, proliferation, metastasis, and chemoresistance [9–11, 45]. At present, the standard chemotherapeutic drugs for NSCLC including first line cisplatin and second line highly selective EGFR-directed tyrosine kinase inhibitors such as gefitinib, erlotinib, and afatinib have been suffering from drug resistance [8, 16]. A large body of literature evidence has shown that all these drugs induce STAT3 activation in NSCLC [15, 16] suggesting that STAT3 might play an important role in the development of chemoresistance to these drugs. To date, several STAT3 inhibitors have been developed that selectively bind with STAT3 thereby inhibiting its phosphorylation and dimerization. However, none of them has been approved for clinical use [46]. Therefore, identification of bioactive molecules able, on one hand, to induce apoptosis in cancer cells and, on the other, suppress STAT3 activation may potentially improve the cancer treatment outcomes. Based on attractive anticancer activity of PSD-A against A549 lung cancer cells, we designed the study to further explore its effect on STAT3 activation. In the present study, we found for the first time that PSD-A in addition to impressive anticancer activity dramatically inhibits both constitutive and inducible STAT3 activations. In line with the inhibitory effect on STAT3 phosphorylation, PSD-A effectively inhibited STAT3 DNA-binding activity. The prosurvival effect of STAT3 activation in A549 cells was validated by the knocking down of STAT3. STAT3 knockdown by shRNA inhibited growth and potentiated PSD-A-induced apoptosis in A549 cells. We have also evaluated the in-depth mechanism by which PSD-A inhibits STAT3 activation in the present study. STAT3 activation is regulated by multiple upstream signaling molecules including JAKs, Src, MAPKs, and PTPs [12]. PTPs such as SHP-1, SHP-2, and PTEN negatively regulate STAT3 activation by dephosphorylating the receptor tyrosine kinases, JAKs, or STAT3 [12]. Although PSD-A increased only SHP-1 expression, the fact that sodium orthovanadate reversed PSD-A-induced inhibition of STAT3 activation suggests that PTPs play a very important role in the inhibition of STAT3 activation by PSD-A. Among nonreceptor tyrosine kinases, JAKs and Src are the main players of STAT3 activation [46]. Indeed, PSD-A decreased the phosphorylation and expression of Src; the phosphorylation of JAK2 remained unaffected. However, momelotinib effectively inhibited STAT3 activation suggesting that JAK-2 activation plays an important role in STAT3 activation in A549 cells. We also showed that although PSD-A increased phosphorylation of JNK, however, it inhibits STAT3 activation independent of JNK activation. Finally, our computational docking study revealed that PSD-A is predicted to bind with the SH2 domain of STAT3 by stable hydrogen bonding which may at least in part be responsible for anti-STAT3 activity of PSD-A. Collective data suggest that PSD-A might inhibit STAT3 activation through multiple mechanisms including direct binding, increased expression of SHP-1, and decreased phosphorylation of Src at least to the extent of this study.

**Figure 8:**
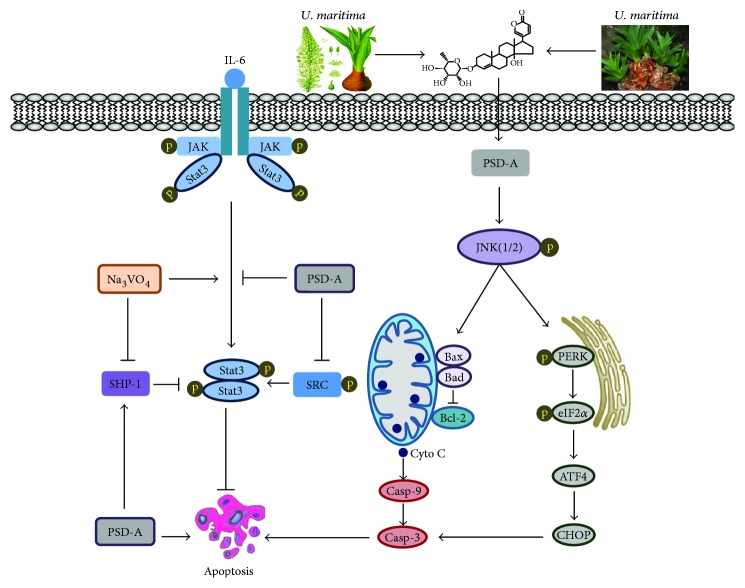
A schematic model for the molecular mechanism of PSD-A-induced anticancer activity in A549 lung adenocarcinoma cells.

In conclusion, we have provided detailed evidence of PSD-A-induced anticancer activity and underlying mechanism in A549 NSCLC for the first time. Based on our experimental data, we have summarized a scheme of the possible mechanism of PSD-A-induced apoptosis in A549 cells (Figure 8). PSD-A inhibited growth and induced apoptosis at an extremely low concentration (25–50 nM). The induction of apoptosis is partly associated with the activation of JNK, induction of ER stress, inhibition of STAT3 activation, and mitochondrial dysfunction. In addition, we provided in-depth molecular mechanism of STAT3 inhibition by PSD-A for the first time in the present study. The data suggest that PSD-A can be developed into a novel STAT3 inhibitor and as a potential anticancer agent against NSCLC.
